# The ctDNA Paradigm: Dynamic Observation, Quantitative Analysis, and Interpretive Limits in Precision Oncology

**DOI:** 10.3390/genes17070754

**Published:** 2026-06-30

**Authors:** Massimiliano Chetta, Nenad Bukvic, Alessandra Rosati

**Affiliations:** 1 Complex Operative Unit of Pathological Anatomy and Molecular Diagnostics, San Giovanni di Dio Ruggi d’Aragona, Schola Medica Salernitana, Largo Città di Ippocrate, 84131 Salerno, Italy; 2 U.O.C. Genetica Medica, Azienda Ospedaliero-Universitaria Consorziale Policlinico di Bari, 70124 Bari, Italy; nenad.bukvic@policlinico.ba.it; 3 Department of Medicine, Surgery and Dentistry, Schola Medica Salernitana, University of Salerno, 84081 Baronissi, Italy; arosati@unisa.it

**Keywords:** ctDNA, clonal hematopoiesis, variant allele frequency, cancer cell fraction, fragmentomics, liquid biopsy

## Abstract

Circulating tumor DNA (ctDNA) was initially conceived as a minimally invasive surrogate for interrogating cancer biology; however, three decades of evidence have demonstrated that plasma is not a passive reservoir of tumor-derived material, but rather a dynamic and biologically heterogeneous milieu in which multiple competing genomic signals coexist. This review explores the level of interpretive rigor required to translate ctDNA detection into clinically actionable precision oncology. Clonal hematopoiesis of indeterminate potential (CHIP) is discussed not as an occasional confounder, but as an intrinsic source of biological background noise, underscoring the critical importance of matched leukocyte sequencing to discriminate tumor-derived alterations from hematopoietic variants, particularly in older individuals and in patients previously exposed to cytotoxic therapies. The widespread assumption that variant allele frequency (VAF) directly reflects tumor burden is critically re-evaluated through the mathematical relationships linking VAF to tumor fraction, local copy-number architecture, and mutation multiplicity. Within this framework, estimation of cancer cell fraction (CCF) and probabilistic discrimination between clonal and subclonal events are examined, including the emergence of reversion mutations as molecular evidence of therapy-driven evolutionary adaptation. The review also addresses the central paradox of ultra-sensitive sequencing technologies: although unique molecular identifiers and duplex sequencing can extend analytical sensitivity below 0.01% VAF, sensitivity in the absence of contextual specificity risks conflating technical artifacts and biologically insignificant alterations with clinically meaningful disease. Equal emphasis is placed on pre-analytical variables, highlighting how sample collection, stabilization, and processing protocols define the upper limit of downstream analytical reliability. Beyond single-nucleotide variants, fragmentomic and methylation-based approaches are presented as complementary orthogonal dimensions capable of revealing tumor-associated signals even when mutational evidence is limited or absent. Longitudinal ctDNA assessment is argued to provide substantially greater biological and clinical insight than isolated static measurements, while robust clinical reporting is shown to depend on transparent disclosure of assay limitations, residual uncertainty related to CHIP, and structured bidirectional communication between molecular laboratories and treating clinicians. Ultimately, the transition from a biomarker-centered model toward an integrated systems-based framework, combining genomics, epigenomics, fragmentomics, and evolutionary modeling, emerges as the defining challenge for the next generation of liquid biopsy in precision oncology.

## 1. Introduction

Three decades ago, drawing upon the first evidence dating back to 1994, when both Vasioukhin et al. and Sorenson et al. independently demonstrated the presence of tumor DNA in blood plasma, the detection of circulating tumor DNA began. At the time, liquid biopsy was perceived as a transformative shortcut toward minimally invasive tumor monitoring. The foundational discovery of that period established that plasma is not merely a transport medium, but an informational archive of tumor physiology and pathology. Liquid biopsy appeared to promise what oncological medicine had always desired: the ability to observe cancer not as a static sample fixed in formalin, but as a living process, a motion picture rather than a photograph [1].

Yet the expansion of analytical capability rapidly revealed previously unrecognized layers of biological complexity. It was soon understood that expanding the power of observation does not simplify reality; rather, it exposes certainty in all its unsettling complexity. Today, we know that the analytical sensitivity of ctDNA, although reaching detection levels on the order of 0.01% variant allele frequency (VAF) through next-generation sequencing (NGS) and digital PCR (ddPCR) techniques [2,3,4], remains inadequate in contexts of low tumor burden, such as early-stage tumors or neoplasms characterized by low molecular shedding. Recent studies demonstrate that in stage I non-small cell lung cancer (NSCLC), detection sensitivity varies significantly depending on the technology employed: whereas conventional assays show limited sensitivity, tumor-informed approaches using multiplex PCR and ultra-deep sequencing have reached markedly improved detection rates in stage I disease, with peaks exceeding 80% in molecular response monitoring contexts [3]. These data highlight that the broad sensitivity range often cited is strongly dependent on the analytical method, sequencing coverage, and histological tumor characteristics (for instance, lung adenocarcinoma shows higher shedding patterns than squamous cell carcinoma), with critical implications for early diagnosis and post-surgical risk stratification [2,5]. The ctDNA signal proved to be embedded within substantial biological noise, technical variability, and dynamic evolutionary processes. Extracting meaning from this signal today requires not only technological precision, but also a conceptual rigor that is still being learned.

## 2. CHIP: The Hidden Biological Noise Within Blood Sample

The first great lesson came from studying a phenomenon initially considered a rare confounder: clonal hematopoiesis of indeterminate potential, or CHIP. Today recognized as a constitutive feature of hematopoietic aging, its discovery came as a surprise when it was realized that DNA detected in plasma, specifically somatic mutations in *DNMT3A* (DNA Methyltransferase 3 Alpha), *TET2* (Tet Methylcytosine Dioxygenase 2), *ASXL1* (Additional Sex Combs Like 1), *TP53* (Tumor Protein p53), and *PPM1D* (Protein Phosphatase, Mg^2+^/Mn^2+^ Dependent 1D), did not always originate from the solid tumor under treatment, but from clonal expansions of hematopoietic stem cells accumulated with age or under the pressure of cytotoxic therapies [6,7,8]. Ultra-deep sequencing analyses provided quantitative evidence of the extent of this phenomenon. In a study integrating matched plasma and leukocyte sequencing, Razavi and colleagues found that variants compatible with clonal hematopoiesis accounted for 81.6% of somatic alterations detected in healthy individuals and for 53.2% of those identified in patients with metastatic cancer, highlighting clonal hematopoiesis as a major contributor to the somatic mutational landscape observed in plasma cfDNA [9].

The formal definition of CHIP requires the absence of morphological criteria for a hematological neoplasm, the exclusion of other clonal conditions (such as PNH (Paroxysmal Nocturnal Hemoglobinuria), MGUS (Monoclonal Gammopathy of Undetermined Significance), MBL (Monoclonal B-cell Lymphocytosis)), and the presence of a somatic mutation associated with hematological neoplasms at an allelic frequency of at least 2% in the hematopoietic compartment; although in plasma, due to the dilution of circulating DNA, such mutations may appear at apparently lower VAFs [6,7].

This discovery fundamentally altered the interpretation of plasma-derived genomic information. Plasma is no longer regarded as a simple genomic space, but rather a composite mosaic, in which circulating tumor DNA (ctDNA) is only one of the narrators. ctDNA is indeed a specific fraction of cell-free DNA (cfDNA), released primarily through apoptotic processes and, secondarily, through tumor cell necrosis. Mathematical modeling suggests that only a minute fraction of a cancer cell’s genome, on the order of hundredths of a percent, is shed into the blood after each apoptotic event, with an estimated plasma half-life ranging from minutes to a few hours [4,10]. This shedding value, derived from models of renal and hepatic clearance, underscores that the quantity of tumor DNA released is a function not only of tumor mass, but also of cellular turnover velocity and removal efficiency by the host organism [10]. This brief half-life confers upon ctDNA the potential to serve as a real-time biomarker, immediately responsive to therapeutic perturbations, but it also implies that every single measurement is merely a frame from a continuously moving film, susceptible to random or systematic variations [8,10].

Somatic alterations progressively accumulate within hematopoietic stem and progenitor cells throughout life, leading to the emergence of expanded blood cell clones in otherwise healthy individuals. This process becomes increasingly common with aging and is further shaped by exposure to anticancer treatments. Population-based sequencing studies have shown that detectable CHIP is uncommon before midlife but rises steeply thereafter, affecting approximately 10–20% of individuals older than 70 years and up to 20–50% of those in the oldest age groups, depending on sequencing depth and variant detection thresholds. Among patients previously treated with cytotoxic therapies, particularly myelosuppressive regimens, clonal hematopoiesis is observed even more frequently [7,9].

The extent to which clonal hematopoiesis interferes with plasma-based genomic profiling is therefore highly dependent on patient characteristics. Advancing age and prior exposure to DNA-damaging agents, especially alkylating compounds and platinum-containing chemotherapy, favor the expansion of hematopoietic clones and increase the likelihood that blood-derived variants will be detected in circulating cell-free DNA. For this reason, concurrent sequencing of peripheral blood leukocytes or buffy coat DNA has become essential for distinguishing hematopoietic mutations from true tumor-derived alterations and for ensuring reliable interpretation of liquid biopsy findings [7,9].

Particularly compelling evidence indicates that cytotoxic treatment exerts a selective pressure on pre-existing hematopoietic clones carrying mutations in DNA damage response genes such as *PPM1D* and *TP53*. Exposure to alkylating agents and platinum-based chemotherapy preferentially promotes the expansion of these mutant clones, generating a therapy-related clonal architecture that may remain detectable long after treatment completion. Consequently, the genomic composition of peripheral blood represents a dynamic and continuously evolving system whose trajectory may be largely independent of that of the solid tumor under investigation [6,7].

The specific clinical issue is that it is no longer possible to automatically assume that a mutation in *BRCA1*, *BRCA2*, *ATM*, or *CHEK2*, genes that direct rapid therapeutic decisions, including the use of PARP inhibitors, is present in the carcinoma. Critically, mutations in *PPM1D* or *TP53* in the blood may instead reflect chronic chemotherapy toxicity rather than incipient acute myeloid leukemia or metastatic carcinoma, complicating interpretation in pretreated patients [6,7]. Such variants often originate from clonal hematopoiesis rather than the solid tumor itself; if not recognized through paired sequencing of peripheral blood mononuclear cells (PBMCs), they could lead clinicians to prescribe targeted therapies against the wrong target. In this context, the 2022 ESMO guidelines underscore the indispensable necessity of comparing the mutational profile of plasma with DNA from leukocytes or buffy coat to exclude artifacts of hematopoietic origin, particularly when testing DNA repair genes (HRR) in solid tumors.

CHIP, therefore, is not an anomaly to be catalogued among rare artifacts; it is a constitutive feature of the aging human genome. It constitutes the biological background noise against which every tumor signal must be decoded, a filter through which one must pass before daring to interpret any variant. The distinction thus requires a mandatory comparison between plasma and the patient’s leukocytes: only variants present in plasma but absent, or at significantly lower frequency, in the hematopoietic compartment can be attributed with reasonable confidence to the solid tumor [6]. This approach, defined as “matched normal sequencing,” represents a standard of care in precision oncology practice today, and its omission constitutes a primary interpretive error.

## 3. VAF: From Raw Measurement to Meaningful Biology

Faced with this complexity, initial recourse was made to an apparently solid anchor: the variant allele frequency (VAF). The proportion of sequencing reads supporting a mutation relative to total coverage provides an apparently straightforward quantitative metric, offering the illusion of precise quantification. But here enters the crucial difference between measurement and interpretation. VAF is, in its essence, a technical parameter: the ratio of mutant reads to total reads at a given locus. It is not, however, a direct measure of tumor burden, clonal dominance, or biological relevance [11,12]. Prognostic studies in large cohorts of patients with metastatic solid tumors have shown that maximum VAF is significantly associated with overall survival in multivariate analysis, but this association reflects the complexity of the clinical picture (visceral metastases, performance status, mutational burden) rather than a simple numerical equivalence with tumor burden [13].

To transform this raw measure into meaning, it must be immersed in a mathematical model that recognizes its nature as a mixture. Circulating DNA is a mixture: one part derives from tumor cells, one part from normal cells. The observed VAF is therefore a function of the tumor fraction in plasma (ft), the number of gene copies in tumor cells (Ct), the copies in normal cells (Cn, typically 2), and the multiplicity of the mutation (m, that is, how many mutant alleles exist at that locus).

The resulting mathematical framework illustrates the composite nature of plasma-derived mutational signals. The expected VAF corresponds to:

VAF = (ft × m)/[ft × Ct + (1 − ft) × Cn]

This equation reveals a fundamental truth: VAF is determined as much by tumor biology as by sample composition. It is therefore not possible to interpret a VAF of 0.5% as “little tumor” and one of 20% as “much tumor” without knowing the genomic context. A CHIP mutation may show an elevated, stable VAF over time precisely because it originates from an expanded hematopoietic clone, whereas a true tumor mutation, in a tumor that sheds little DNA or in an early phase, may oscillate at the limits of detectability. It is thus indispensable to integrate VAF with the estimate of global tumor fraction to avoid misleading interpretations [9,11].

For this reason, tumor fraction estimation requires sophisticated strategies that surpass the fragile heuristic based on maximum VAF. Alternative approaches such as the Tumor Fraction Estimator (TFE), based on measuring tumor aneuploidy through copy number alteration (CNA) analysis, and the Maximum Somatic Allele Frequency (MSAF) have been proposed. However, while TFE is more robust to CHIP interference, MSAF, based on the maximum detected VAF, does not account for this source of biological noise, rendering it less reliable in elderly or pretreated patients [11]. Bio-physical approaches such as ichorCNA (integrated analysis of copy number alterations in ultra-low-pass whole-genome sequencing data), which estimate tumor fraction through whole-genome sequencing at low coverage (0.1× WGS) and analysis of aneuploidy profiles, represent the current frontier for genotype-independent quantification, particularly useful when the mutational signal is absent or ambiguous [12]. The most common heuristic, albeit fragile, uses the maximum observed VAF: assuming that the most frequent mutation is clonal and heterozygous, one posits ft ≈ 2 × VAFmax. “However, this approximation becomes unreliable when CHIP mutations are erroneously included, or when the tumor harbors extensive amplifications or deletions. More robust approaches rely on genomic profiles of copy number alteration (CNA), calculating the observed deviation from diploidy relative to the expected tumor signal, or on fragmentomic and methylation analyses, which in low-shedding tumors can reveal tumor presence even when the mutational signal is absent or too weak [9,13].

From this emerges the conceptual distinction between VAF and MAF (Mutant Allele Fraction) in the interpretive sense. While VAF is what is experimentally measured, MAF represents the estimate of the fraction of mutant alleles actually derived from tumor cells. To obtain it, one must correct VAF for tumor fraction and copy number alterations. Under restrictive assumptions (absence of copy number alterations, heterozygosity, no allele loss) it is possible to roughly estimate tumor fraction as ft ≈ 2 × VAF/m. Yet such assumptions rarely hold in clinical reality. Tumors are frequently characterized by aneuploidy, subclonal diversification, and continuous evolutionary adaptation.

In the case of homozygosity (or when the mutation is present on both alleles in tumor cells), the standard assumption ft ≈ 2·VAF becomes erroneous and leads to a 100% overestimation of tumor fraction (Table 1) [12].

For the laboratory, there are several implications into which one may run:

Systematic error: Erroneously assuming heterozygosity for a homozygous mutation yields a tumor fraction estimate double the reality (e.g., a patient with ft 10% shows VAF 10% for a homozygous mutation, and one erroneously calculates ft 20%) [12].

Modes of distinction in allelic ratio analysis: In pure homozygosity, the mutant/wild-type ratio tends toward infinity (absence of wild-type reads), whereas in heterozygosity it is 1:1 (barring LOH). One must systematically verify the absence of reference (wild-type) reads on both strands to support the homozygosity hypothesis, using duplex sequencing approaches that reduce alignment artifacts. One must verify whether deletions or amplifications of the locus exist through copy number alteration (CNA) analysis. A heterozygous deletion suggests LOH. Compare the VAF of the target mutation with other clonal mutations. If mutation “A” has twice the VAF of mutation “B” (both clonal), “A” is likely in homozygosity or in an amplified region [10]. When copy state is uncertain, it is prudent to report a range: “Estimated tumor fraction 10–20%, assuming homozygosity or heterozygosity of the dominant clonal mutation, respectively.”

If the tumor presents LOH (loses the WT allele) and the mutation is homozygous (duplication of the mutant allele or biallelic mutation), then Ct = 2 but both alleles are mutated (m = 2), still leading to ft ≈ VAF [12].

The interpretive challenge intensifies when one considers the stochastic variability of VAF. At low allelic frequencies, statistical sampling becomes dominant: the binomial distribution of reads means that a VAF of 0.1% with 10,000× coverage has a wide confidence interval. The variance of the binomial estimate implies that, at equal VAF, a coverage of 30,000× significantly reduces the confidence interval compared with a coverage of 1000×, making longitudinal monitoring more reliable when platforms with high sequencing depth are used.

At low allelic frequencies, distinguishing true biological variation from stochastic sampling noise becomes statistically challenging. Because only a minute fraction of tumor DNA enters circulation per cell death in tumors smaller than 1 cm^3^, where tumor fraction is estimated at merely 0.022%, a 15 mL blood sample may contain on average only 1.7 haploid genome equivalents (hGE) of tumor origin, making detection highly stochastic. In these contexts of extreme signal rarity, the law of large numbers does not apply, and the Poisson distribution better describes the probability of detection, which becomes strongly dependent on the volume of blood drawn and the processing time of the sample [9].

This sampling limitation motivates a broader methodological consideration. As ctDNA assays become increasingly sophisticated, the trade-off between sequencing breadth and depth has emerged as a central methodological issue. Targeted approaches achieve very high coverage across selected genomic loci, often exceeding 10,000×, thereby maximizing analytical sensitivity for predefined alterations. Nevertheless, when ctDNA is present at extremely low levels, these assays remain susceptible to sampling limitations because mutant fragments may simply be absent from the aliquot analyzed. Genome-wide strategies address this constraint by integrating information across large numbers of somatic alterations distributed throughout the tumor genome, thus reducing the influence of stochastic sampling effects. Tumor-informed whole-genome approaches combined with machine-learning algorithms, such as MRD-EDGE, further enhance this concept by exploiting ctDNA-specific molecular features and deep-learning models to enrich true variant signals. In validation studies, MRD-EDGE increased SNV signal enrichment by approximately 300-fold compared with earlier WGS-based error suppression methods and enabled detection of tumor fractions below 0.001%, supporting highly sensitive plasma-only disease monitoring. These developments suggest that comprehensive genomic interrogation, when coupled with advanced computational integration, may overcome some of the intrinsic limitations of narrowly focused ultra-deep sequencing [14].

Finally, copy-number alterations provide an additional and largely independent source of tumor-derived information in plasma. Given that chromosomal instability and aneuploidy are common features of many solid malignancies, genome-wide assessment of CNAs offers a mutation-agnostic strategy for estimating tumor fraction. Recent machine-learning-based analyses applied to shallow whole-genome sequencing data have markedly improved analytical performance, lowering the amount of detectable aneuploid genome required for ultrasensitive ctDNA identification from approximately 1 Gb to 200 Mb. Moreover, tumor-informed CNA profiling approaches, such as informCNA, have demonstrated reliable ctDNA detection at tumor fractions as low as 0.2% and, in ovarian cancer cohorts, identified disease recurrence several months earlier than conventional serum biomarkers. Collectively, these observations indicate that CNA analysis should not be regarded only as ancillary to SNV detection, but rather as a complementary and, in specific clinical settings potentially superior, measure of residual disease and tumor burden [15].

## 4. Clonal Architecture and the Elusive Cancer Cell Fraction (CCF)

Yet understanding the quantity of tumor in the blood is only the beginning. It is necessary to understand who that tumor is. Cancer represents a heterogeneous evolutionary system composed of dynamically competing cellular populations, governed by the laws of natural selection applied at the molecular scale. Liquid biopsy, sampling DNA from all these populations simultaneously, offers a composite portrait of this diversity, but with a profound spatial ambiguity: one does not know from which anatomical site each DNA fragment originates.

The crucial distinction is between clonal mutations, present in the majority of tumor cells and representing foundational events of oncogenesis, and subclonal mutations, which define emerging or minor populations, often carrying mechanisms of resistance or adaptation. To quantify this aspect, one turns to the Cancer Cell Fraction (CCF), which expresses the proportion of tumor cells carrying a given mutation. Its mathematical formulation corrects VAF for tumor fraction, copy number, and multiplicity:

CCF = [VAF × (ft × Ct + (1 − ft) × 2)]/(ft × m)

When CCF approximates unity, one speaks of a clonal mutation; when lower, of a subclonal one. The distinction between clonal and subclonal alterations should be interpreted as probabilistic rather than strictly binary: mutations with CCF between 0.8 and 1 may represent dominant but not universal clones, whereas CCF values < 0.5 clearly indicate subclonality [11].

However, even this distinction requires extreme caution. The general formulation of CCF requires solving constrained optimization problems, often addressed through Bayesian mixture models that estimate the probability of a mutation belonging to specific clones. But in plasma, signal dilution, measurement noise, and the overlap of VAF distributions dramatically limit the resolution of these models. The statistical identifiability of CCF remains problematic when mutations coexist in regions with copy number alterations (CNA), requiring evolutionary assumptions such as the “single split copy number assumption” to constrain the space of possible genotypes [11]. Recent algorithms such as DeCiFer propose the use of Descendant Cell Fraction (DCF), a generalization of CCF that accounts for mutation losses during clonal evolution, offering more parsimonious phylogenetic reconstructions. DeCiFer overcomes the limitations of conventional methods (such as PyClone) that assume constant mutation multiplicity, enabling correct grouping of mutations that, despite having different VAFs due to subsequent deletions or amplifications, belong to the same foundational clonal event [15,16].

Furthermore, the distribution of VAFs across mutations can suggest clonal architecture: clusters of mutations with similar VAFs suggest co-membership in the same clone, whereas clusters at lower VAFs indicate subclones. Bayesian mixture algorithms can estimate the probability that a mutation belongs to a given clone. Nevertheless, reconstructed clonal architectures frequently remain partially unresolved due to signal overlap, copy number complexity, and plasma dilution effects, an approximation of a more complex reality where distinct subclones may merge into a single indistinguishable cloud of VAFs.

In this context, it becomes fundamental to distinguish driver mutations, which confer selective advantage and shape tumor behavior, from passenger mutations, accumulated stochastically. Paradoxically, it is often the passengers, numerically dominant, that define tumor mutational burden and contribute to neoantigen formation, even if they do not directly drive growth. Liquid biopsy imposes holding these contradictory truths together: the biological meaning of a mutation is inseparable from its temporal and evolutionary context. A subclonal mutation today could become the dominant clonal mutation after three months of selective therapy. Therapy-induced clonal selection can indeed expand pre-existing or de-novo resistant populations, making serial monitoring of clonal architecture an indispensable requirement for dynamic precision medicine [16].

## 5. Reversion Mutations: When Cancer Learns to Outsmart Therapy

Among the most compelling manifestations of therapy-driven tumor evolution are reversion mutations, secondary genetic events that restore the function of genes previously rendered inactive by pathogenic alterations. These rescue events may arise through secondary point mutations, small insertions or deletions, larger genomic rearrangements, or recombination-mediated mechanisms, ultimately re-establishing protein function and conferring therapeutic resistance.

Mechanisms of acquired resistance have been particularly well characterized in the setting of *EGFR*-mutant non-small cell lung cancer. Following treatment with first- and second-generation *EGFR* tyrosine kinase inhibitors, the p.T790M substitution in *EGFR* emerges as the predominant resistance mechanism, increasing ATP affinity and thereby diminishing inhibitor binding. Subsequent exposure to osimertinib frequently selects for additional escape mechanisms, most notably the *EGFR* p.C797S mutation, often accompanied by MET amplification or histologic transformation. Together, these alterations exemplify the stepwise selective pressures imposed by targeted therapies and illustrate how resistant subclones evolve during treatment [17,18].

True reversion mutations have been most extensively investigated in tumors harboring pathogenic alterations in *BRCA1* or *BRCA2*. Deficiency of homologous recombination repair sensitizes these cancers to platinum compounds and PARP inhibitors. Under therapeutic pressure, however, tumor cells may acquire secondary genetic alterations that restore the open reading frame and re-establish homologous recombination proficiency. Functional restoration of BRCA proteins consequently reduces sensitivity to both platinum-based chemotherapy and PARP inhibition. Recent studies in metastatic prostate cancer have further shown that *BRCA1/2* reversion mutations may emerge even in the absence of prior PARP inhibitor exposure, following treatment with platinum agents or docetaxel, suggesting that conventional systemic therapies may contribute to the development of primary resistance to subsequent targeted approaches [16].

From an analytical perspective, reversion mutations pose substantial challenges. They are typically detected at very low allele frequencies, often coexist with the original pathogenic variant, and may arise independently in distinct subclonal populations, a phenomenon referred to as convergent polyclonality. Their identification therefore requires highly sensitive assays capable of resolving sequence changes at specific genomic loci, together with longitudinal sampling to monitor the temporal relationship between the founding alteration and the emerging reversion event. Progressive increases in the ratio between reversion and founder mutation VAFs during treatment provide evidence of selective expansion of resistant clones.

The clinical value of this approach was elegantly illustrated in *BRCA2*-associated pancreatic cancer, where ctDNA analysis enabled the identification of a *BRCA2* p.S2835L reversion mutation following resistance to FOLFIRINOX therapy. This observation demonstrated that liquid biopsy can capture clonal evolution in real time, even outside the context of PARP inhibitor exposure [19]. In this regard, ctDNA analysis has evolved from a purely descriptive biomarker into a dynamic predictive tool, capable of revealing resistance mechanisms while they are emerging and potentially opening a therapeutic window before overt radiological progression becomes evident.

## 6. Ultra-Sensitivity Is Not Enough: The Trap of Analytical Precision

Modern sequencing technology has reached levels of sensitivity through the use of unique molecular identifiers (UMIs) and duplex sequencing, where both DNA strands are sequenced and reads are compared to eliminate polymerase errors; error rates have been reduced to levels of one in a million, theoretically allowing detection of variants at frequencies below 0.01%. Yet, the clinical implementation of such technologies requires rigorous quality control: duplex sequencing, for example, requires that both strands carry the same variant for the call, drastically reducing false positives but increasing DNA input requirements and the effective sequencing depth needed. This technological progress introduces a major interpretive risk: confusing analytical sensitivity with clinical reliability.

It must be remembered that sensitivity is not an absolute property; it depends on sequencing quality, coverage depth, and error suppression. In real-world contexts, background noise remains substantial, particularly in genomic regions prone to sequencing artifacts (pseudogenes, repetitive regions, homopolymers). The relationship between Phred quality and error probability (where Q30 indicates one error in a thousand, Q40 one in ten thousand) reminds us that there exists a physical limit beyond which signal merges with noise [18,19,20]. It is fundamental to understand that library preparation errors (for instance, cytosine-to-thymine deamination) can dominate background noise at Q40 quality, making the limit of detection (LoD) often determined more by pre-analytical and library artifacts than by pure instrumental accuracy [19,20].

Moreover, increased sensitivity inevitably leads to the detection of spurious or biologically irrelevant signals. A variant detected at 0.05% does not guarantee clinical significance. It could be a mutation present in a single hematopoietic cell, a sample preparation artifact, or a sequencing error surviving correction. Clinical utility depends not only on analytical sensitivity but also on robust interpretive specificity capable of discriminating biologically meaningful variants from technical artifacts. This distinction requires knowledge of the limit of detection (LoD), which must always be greater than or equal to the background error rate, and a deep understanding of biological context. Accurate interpretation therefore requires stringent filtering strategies and rigorous biological contextualization, in recognizing that informational silence is preferable to deceptive noise. ESMO guidelines explicitly recommend that every ctDNA report include the LoD specific for each target variant and the method’s background error rate, to enable appropriate clinical evaluation [2].

The problem of inter-laboratory standardization is critical. The lack of uniform protocols for the pre-analytical, analytical, and post-analytical phases generates variability in results that precludes reliable multicenter comparisons. External quality assurance programs and proficiency testing remain imperative for routine implementation. Adherence to accreditation criteria (ISO 15189, CAP/AMP) and participation in external quality assessment (EQA) programs represent non-negotiable prerequisites for the clinical use of ctDNA assays, as underscored by recent international recommendations [2].

## 7. Garbage in, Garbage out: The Critical Role of Pre-Analytical Variables

Pre-analytical variables substantially determine downstream sequencing quality and interpretive reliability. The manner in which blood is drawn, the time elapsed before centrifugation, the protocol used to separate plasma and the prevention of cellular contamination, every single step influences the composition of the extracted DNA. The use of tubes specifically designed for cfDNA stabilization (such as Streck Cell-Free DNA BCTs, which contain formaldehyde and stabilizing buffers) can prevent leukocyte lysis and DNA degradation for up to 7 days at room temperature, although processing within 4 h of collection remains the gold standard, particularly for samples in conventional EDTA tubes [11]. It is equally important to use purification kits validated for compatibility with the specific blood collection tube chemistry, as extraction efficiency and fragment-size bias vary considerably across platforms; appropriately matched workflows improve the recovery of circulating tumor DNA and reduce the carryover of genomic DNA from lysed cells.

An inadequately processed sample may undergo lysis of residual leukocytes in plasma, releasing genomic DNA that dilutes the ctDNA fraction and introduces confounding signals, especially CHIP mutations residing in leukocytes. Hemolysis or incomplete plasma separation can measurably alter circulating DNA characteristics, also modifying fragmentation patterns that one wishes to study. These variables are fundamental determinants of assay performance and reproducibility; they are fundamental determinants of data quality. Measurement precision begins with preparation precision, with the awareness that the sample is fragile, that circulating DNA has a short half-life, and that every minute of delay or every handling error contaminates the final interpretation.

Standardization of collection protocols, the use of specific stabilizing tubes (containing formaldehyde or other agents that preserve cellular integrity while preventing lysis), and rigorous training of laboratory personnel are non-negotiable investments. Without this solid foundation, even the most sophisticated broad-panel or single-molecule sequencing is destined to produce unreliable data. Double-spin centrifugation (first at low speed to separate plasma, then at high speed to remove residual cells) is recommended to obtain truly cell-free plasma and minimize genomic DNA contamination [2].

## 8. Beyond Mutations: Fragmentomics and Methylation as New Frontiers

Early liquid biopsy approaches focused predominantly on somatic point mutation detection: one sought point mutations, nucleotide alterations. Yet the DNA circulating in blood is an archive of far richer information. DNA fragmentation modalities reflect chromatin structure and nucleosome positioning of the tissue of origin. Tumor DNA tends to fragment into shorter pieces, around 100–150 base pairs, compared with the peak at approximately 167 base pairs of non-tumor DNA, deriving from regular nucleosomal cleavage by DNase in healthy cells [12].

These fragmentation patterns provide an independent structural layer of tumor-associated information, a structural signature of the tumor. The ratio between short fragments and mononucleosomal fragments (those around 167 bp) correlates with tumor DNA presence and can be integrated into tumor fraction estimation models, particularly useful when the mutational signal is absent or ambiguous. Analysis of end motifs, fragmentation peaks, and cutting preferences offers further informative dimensions. Shannon entropy of fragments at the first exon level (E1SE) can differentiate specific tumor subtypes, such as lung adenocarcinoma from squamous cell carcinoma, with an AUC of 0.90, or identify androgen receptor activity in prostate cancer, offering a non-invasive surrogate of tissue-specific gene expression [12].

The integration of fragmentomic data with machine learning algorithms (ichorCNA for tumor fraction estimation) enables detection of tumor presence even when the mutational signal is absent or too weak to be detected conventionally. In particular, methods such as SRFD (Semi-Reference-Free Deconvolution) and CelFiE allow estimation of proportions of known and unknown cell types from cfDNA methylation profiles, overcoming the need for reference tissue biopsies. These probabilistic deconvolution algorithms exploit the fact that cfDNA methylation patterns are a mixture of epigenetic signatures from all tissues contributing to the circulating pool, enabling attribution of DNA origin even in the absence of a tumor reference sample [12].

DNA methylation profiling further expands the diagnostic and tissue-classification capabilities of cfDNA analysis. Methylation patterns provide highly informative signatures, capable of identifying the tissue of origin of circulating DNA and of detecting cancers at early stages, when driver mutations are still rare or absent. Methylation is tissue-specific and systematically altered in cancer (global hypomethylation and selective promoter hypermethylation). The integration of these genomic, epigenomic, and structural dimensions through machine learning algorithms and probabilistic models represents the current frontier [19,20]. Methylation-based classifiers, such as those employed for cancers of unknown primary (CUP), achieve accuracies above 80% in identifying the primary tissue, and when integrated with ichorCNA for tumor fraction estimation, they allow detection of tumor burdens below 3%, below which deconvolution becomes statistically unstable [21].

Recent advances in chemical sequencing strategies are progressively narrowing the gap between genetic and epigenetic profiling. Methods such as TET-assisted pyridine borane sequencing (TAPS) and related conversion chemistries have been developed as alternatives to bisulfite treatment, enabling the interrogation of cytosine methylation while largely preserving DNA integrity and improving mapping efficiency. By converting methylated cytosines into thymine analogues while leaving unmethylated cytosines unmodified, these approaches reduce sequence degradation and mitigate the compositional bias typically associated with bisulfite-based protocols. In addition to improved read alignment and reduced data loss, TAPS-based workflows have been reported to streamline downstream computational processing compared with conventional bisulfite sequencing pipelines, although the magnitude of these gains remains dependent on assay design and analytical framework. Within low-input applications, including circulating cell-free DNA, these chemistries have been applied successfully at nanogram-scale inputs, supporting integrated analyses that combine methylation profiling, fragment-level features, and somatic variant detection within a single experimental workflow, primarily in proof-of-concept settings [22,23].

Beyond plasma, the diagnostic performance of circulating tumor DNA is strongly influenced by the anatomical compartment from which biofluids are derived. In central nervous system malignancies, cerebrospinal fluid has consistently shown higher tumor DNA fractions and improved concordance with matched tumor tissue compared with plasma, reflecting the restrictive effect of the blood–brain barrier on ctDNA release into the circulation [24]. In pancreatic neoplasia, cyst fluid represents a direct source of molecular information from precursor lesions, where recurrent mutations such as those in *KRAS* and *GNAS* provide clinically relevant diagnostic support in mucinous cystic entities [25]. Similarly, urine has emerged as a non-invasive medium for ctDNA and extracellular vesicle analysis in urothelial and other genitourinary cancers, with growing evidence supporting its utility for molecular characterization and disease monitoring, although its role in early detection remains investigational. Collectively, these observations underscore that the optimal choice of biofluid is context-dependent and should be guided by tumor biology and anatomical accessibility rather than a plasma-centric paradigm alone [26].

## 9. Tracking Tumors over Time: The Power of Longitudinal Monitoring

The half-life of ctDNA, which ranges from a few minutes to hours (estimated between 30 min and 2 h in studies with renal and hepatic clearance), is simultaneously its greatest gift and its fundamental limitation [9]. The short plasma half-life of ctDNA enables near real-time assessment of tumor dynamics, an indicator immediately responsive to therapeutic perturbations.

To understand a tumor’s behavior, one must observe it over time. Longitudinal sampling allows recognition of trends, capture of emerging resistances, and assessment of therapeutic responses with sensitivity superior to conventional radiological imaging. It transforms liquid biopsy from a static, diagnostic test into a dynamic monitoring tool. The clinical value of longitudinal monitoring emerges primarily from temporal trajectory analysis rather than isolated measurements: a VAF that decreases under therapy tells a different story than a stable or rising VAF, even if the absolute value is identical. ESMO guidelines specifically recommend the use of longitudinal monitoring for molecular response assessment, suggesting sampling intervals varying from 4 to 8 weeks depending on clinical context and tumor type. Temporal integration is mathematically expressed in the comprehensive tumor signal function, which depends not only on VAF, CNAs, and fragmentation, but also on time: Signal(t). Post-surgical ctDNA clearance, for example, is a powerful predictor of long-term outcome; its persistence indicates residual disease [2,3].

## 10. Reporting with Rigor: From Bioinformatics to Bedside Communication

The analytical and interpretive complexity of ctDNA testing ultimately converges during clinical reporting, when the molecular biologist translates sequencing bytes into words that will guide a clinical decision. This step, often underestimated, is perhaps the most delicate phase of the entire process. A universal standard for ctDNA reporting does not yet exist, and the heterogeneity of platforms, gene panels, and analysis algorithms makes standardization a distant goal. The ESMO guidelines represented a first attempt at harmonization, defining tumor-specific recommendations and levels of evidence, but reporting remains a process requiring multidisciplinary skills and integration into clinical context [2]. Yet several fundamental principles must guide the modern laboratory.

First, reporting must begin with rigorous assessment of sample and data quality. One must verify that the fragmentation profile is consistent with circulating DNA (peak at 160–170 bp), excluding significant genomic DNA contamination (peak at >10,000 bp or high-molecular-weight smearing). It must also be confirmed that sequencing coverage is adequate for target variants (typically >1000× for low-frequency variants, >10,000× for ultra-sensitive monitoring) and that process controls (no-template controls, known positive samples) have passed. The inclusion of process controls in every sequencing run, including reference samples with certified VAF at 0.1% and 1%, is essential to guarantee metrological traceability of the assay [3].

The next step is aggressive yet intelligent bioinformatic filtering. Every detected variant must be compared against databases of common germline variants (gnomAD, ExAC) to exclude polymorphisms; against databases of technical artifacts (blacklist regions of problematic genomic regions); and, crucially, against sequencing of the patient’s hematopoietic cell DNA (PBMC or buffy coat). Only variants absent or at significantly lower frequency in the hematopoietic compartment can be considered ctDNA candidates [6]. The practical threshold for defining a variant as “significantly lower” in the hematopoietic compartment is typically set at <5% of plasma VAF or a plasma/PBMC ratio > 5, although these cut-offs remain subject to standardization [2].

For candidate variants, the laboratory must calculate VAF, the statistical confidence interval, and evaluate read quality (Phred score, strand bias, presence of PCR artifacts). The report must then include a tumor fraction estimate. If the panel includes copy number alteration (CNA) regions, a quantitative indication of tumor burden based on coverage deviations from normal can be provided. If bio-physical approaches (fragmentomics) are used, the anomalous fragmentation ratio must be indicated. This quantitative information is essential to contextualize molecular variants: a VAF of 0.5% in a patient with ft of 10% has different implications than the same VAF in a patient with ft of 0.1% [10,11]. Reporting must obligatorily include total allelic coverage (depth), number of mutant reads, mean variant Phred score, and strand bias rate, parameters that allow the clinician to independently evaluate the robustness of the call.

In the interpretive section of the report, the biologist must proceed to classify variants according to actionability and clonality. Clonal variants (CCF near 1) in actionable driver genes must be highlighted as therapeutic priorities. Subclonal variants (CCF < 0.5) require caution: they may represent emerging resistances or spatial heterogeneity. If reversion mutations are detected, these must be correlated temporally with ongoing therapy and compared with any previous tissue biopsies [3,10]. Variants of uncertain significance (VUS) must be classified according to ACMG/AMP criteria adapted for the cfDNA context, with explicit recommendation for re-evaluation in a multidisciplinary setting (molecular tumor board) [2].

The report must always include a limitations section, transparent and honest: indicating the limit of detection (LoD) of the test for that specific variant (e.g., “Variants detectable at frequency > 0.1% with >95% reliability”), warning that possible CHIP presence is not completely excluded, and specifying that a negative result does not exclude disease (false negatives due to low shedding or low tumor fraction). Longitudinal monitoring should also be suggested: “Repeat testing in 4–6 weeks for dynamic confirmation” or “Compare with pre-therapy baseline”. In accordance with ESMO recommendations, the report should distinguish between Tier I variants (actionable with Level I–II clinical evidence) and Tier II variants (actionable in experimental contexts or with limited evidence), facilitating the translation from molecular data to therapeutic decision [2].

Finally, reporting requires bidirectional communication with the clinician. Effective ctDNA reporting requires continuous interaction between molecular laboratories and multidisciplinary clinical teams: the molecular biologist, molecular geneticist, or geneticist must be available to discuss borderline cases, interpret ambiguous results (such as variants of uncertain significance, VUS) in the specific clinical context, and participate in multidisciplinary tumor boards. Only through this tight integration between laboratory and clinic can liquid biopsy express its full potential, preventing raw data, however technically accurate, from generating confusion rather than clarity [3]. Creating an interpretive bridge between the genomics laboratory and the clinical team, through structured reports and active participation in molecular tumor boards, represents the last mile for effective implementation of ctDNA-based precision medicine [2].

## 11. Conclusions

Liquid biopsy today stands at a critical crossroads. Its technological foundations are increasingly robust, and its clinical applicability continues to expand across multiple oncological settings. Yet its interpretation remains, in many respects, immature. The challenges faced are not merely technical; they are conceptual. Precision oncology demands a rethinking of what it means to detect a mutation, quantify a signal, define a biomarker. The naivety of simplistic interpretations must be abandoned in favor of a conceptual framework that makes room for biological complexity, evolutionary dynamics, technical limits, and the integration of multidimensional data. Importantly, VAF should not be interpreted as a direct surrogate for tumor burden, molecular detection does not inherently establish clinical diagnosis, and analytical sensitivity does not necessarily equate to biological certainty [10,11]. The transition from a biomarker-centric paradigm to a systemic paradigm, integrating genomics, epigenomics, fragmentomics, and clinical data into multivariate predictive models, represents the true challenge for the next decade of precision oncology [21].

Only by embedding quantitative measurements within the broader biological context, through mathematical models that recognize the mixed nature of circulating DNA, correction for copy number alterations, and rigorous distinction between tumor signal and CHIP biological noise and only through rigorous, contextualized, and “humble” reporting (in recognizing its own limits), we may begin to get close to a realistic depiction of tumor reality. Rather than simplifying tumor biology, liquid biopsy exposes its evolutionary and spatial complexity with unprecedented resolution. The responsibility falls upon us to develop the conceptual and mathematical tools necessary to interpret that complexity with rigor and precision. It is not a matter of reducing cancer to a number, but of expanding the intelligence available to understand a biological system that, like the blood that hosts it, is in perpetual, elusive motion.

## Figures and Tables

**Table 1:** Estimation of tumor fraction as a function of genetic state.

Genetic State	Ct	m	Formula for ft	Example (VAF = 20%)
Normal heterozygote	2	1	ft ≈ 2·VAF	ft = 40%
Homozygote	2	2	ft ≈ VAF	ft = 20%
LOH (WT deletion)	1	1	ft = 2 × VAF/(1 + VAF)	ft = 33%
Amplification (3 copies, 1 mutated)	3	1	ft = 2 × VAF/(1 − VAF) *	ft = 50% *

* for VAF < 33%.

## Data Availability

No new data were created or analyzed in this study. Data sharing is not applicable to this article.
